# A fiber optoacoustic guide with augmented reality for precision breast-conserving surgery

**DOI:** 10.1038/s41377-018-0006-0

**Published:** 2018-05-18

**Authors:** Lu Lan, Yan Xia, Rui Li, Kaiming Liu, Jieying Mai, Jennifer Anne Medley, Samilia Obeng-Gyasi, Linda K. Han, Pu Wang, Ji-Xin Cheng

**Affiliations:** 1 Department of Biomedical Engineering, Boston University, 44 Cummington Mall, Boston, MA 02215 USA; 2 Vibronix, Inc., 1281 Win Hentschel Boulevard, West Lafayette, IN 47906 USA; 3 Department of Biomedical Engineering, Purdue University, 206 S. Martin Jischke Drive, West Lafayette, IN 47907 USA; 4 Department of Precision Instrument, Tsinghua University, Beijing, 10084 China; 5 Department of Radiology and Imaging Sciences, Indiana University School of Medicine, 550 N. University Blvd, Indianapolis, IN 46202 USA; 6 Department of Surgery, Indiana University School of Medicine, 545 Barnhill Drive, Indianapolis, IN 46202 USA; 7 Parkview Cancer Institute, 11109 Parkview Plaza Drive, Fort Wayne, IN 46845 USA; 8 Department of Electrical & Computer Engineering, Boston University, 8 Saint Mary’s Street, Boston, MA 02215 USA

## Abstract

Lumpectomy, also called breast-conserving surgery, has become the standard surgical treatment for early-stage breast cancer. However, accurately locating the tumor during a lumpectomy, especially when the lesion is small and nonpalpable, is a challenge. Such difficulty can lead to either incomplete tumor removal or prolonged surgical time, which result in high re-operation rates (~25%) and increased surgical costs. Here, we report a fiber optoacoustic guide (FOG) with augmented reality (AR) for sub-millimeter tumor localization and intuitive surgical guidance with minimal interference. The FOG is preoperatively implanted in the tumor. Under external pulsed light excitation, the FOG omnidirectionally broadcasts acoustic waves through the optoacoustic effect by a specially designed nano-composite layer at its tip. By capturing the acoustic wave, three ultrasound sensors on the breast skin triangulate the FOG tip’s position with 0.25-mm accuracy. An AR system with a tablet measures the coordinates of the ultrasound sensors and transforms the FOG tip’s position into visual feedback with <1-mm accuracy, thus aiding surgeons in directly visualizing the tumor location and performing fast and accurate tumor removal. We further show the use of a head-mounted display to visualize the same information in the surgeons’ first-person view and achieve hands-free guidance. Towards clinical application, a surgeon successfully deployed the FOG to excise a “pseudo tumor” in a female human cadaver. With the high-accuracy tumor localization by FOG and the intuitive surgical guidance by AR, the surgeon performed accurate and fast tumor removal, which will significantly reduce re-operation rates and shorten the surgery time.

## Optoacoustics: tumor localization

Implanting an optoacoustic beacon into breast tumors provides surgeons with sub-millimeter localization accuracy when performing surgery. The system has been developed by Lu Lan from Boston University and coworkers. A fiber optoacoustic guide(FOG) which has a fiber tip coated with ZnO nanoparticles and graphite so that it is strongly light absorbing is implanted into the tumor prior to surgery. When exposed to pulsed light excitation the tip generates omnidirectional ultrasound waves due to the optoacoustic effect. The use of three ultrasound sensors to collect this signal makes it possible to accurately triangulate the exact location of the tumor during surgery. Combining the FOG with an augmented reality system offers surgeons an intuitive guidance aid which not only allows accurate and rapid tumor removal but will also help reduce the need for re-operation.

## Introduction

In the United States, breast cancer ranks second as a cause of cancer-related death in women^1^. Breast-conserving surgery (BCS), or lumpectomy, is a standard surgical treatment for early-stage breast cancer and is favored for preservation of the breast, reduced morbidity, and rapid recovery^2–4^. Although BCS is the preferred surgical treatment for early-stage breast cancer, it can be challenging to accurately identify the tumor site when the tumor is small and nonpalpable^5^. This difficulty can lead to inadequate clearance of the margins associated with increased risk of local recurrence, and it requires a second operation for adequate margins. The current re-operation rate is approximately 25% on average, but some series report rates as high as 37%^6–10^. Thus, surgical guidance tools that can accurately locate the tumor and optimize the resection of adequate margins in real time are needed.

Mammogram and magnetic resonance imaging are used to diagnose and localize the breast tumor mass, but these technologies are bulky in size and not accessible in the operating room. The current gold standard of clinical practice to help locate the tumor during the surgery is guide wire localization (GWL) prior to surgery, in which a thin wire is inserted into the tumor mass under image guidance with its terminal tip within the tumor or tumor bed^11^. However, the location of the guide wire tip inside the breast tissue is not visible to the surgeon and only provides a rough estimation of the tumor location. Even with the GWL method, the re-operation rate remains high^12,13^. A newer method is seed implantation to improve the localization of the tumor during the operation. By utilizing a handheld probe to detect a beacon that is pre-implanted in the tumor mass, such as a radioactive/radiofrequency seed^14,15^ or a radiofrequency identification tag (RFID)^16^, the tumor location can be more accurately identified. The radioactive/radiofrequency seed methods can generally provide only the distance of the probe to the beacon in terms of pseudo numbers without units, which is a qualitative measure and is not accurate enough. Although the RFID method can provide quantitative tumor location information, it has a low accuracy of 15 mm. Light-guided lumpectomy, which employs an optical fiber to deliver light to form a visible glowing ball in the breast tissue, has also been developed. However, its detection accuracy is limited and is dependent on the depth of the implanted location (~9% of the depth)^17^. Thus, all current technologies, clinical or preclinical, are limited in localizing the tumor with sufficient accuracy. Moreover, as the detection probe is a separate device from the dissecting instrument, frequent switches between the excision device and the detection probe occur during the surgery, which interferes with the flow and prolongs the surgical time. Furthermore, with continued improvement in imaging technology, smaller lesions are being diagnosed, which increases the demand for high-precision localization^18^. These limitations highlight an unmet need for a precise surgical guidance tool that locates a small tumor with high accuracy and minimal disruption of the surgical flow.

**Fig. 1:**
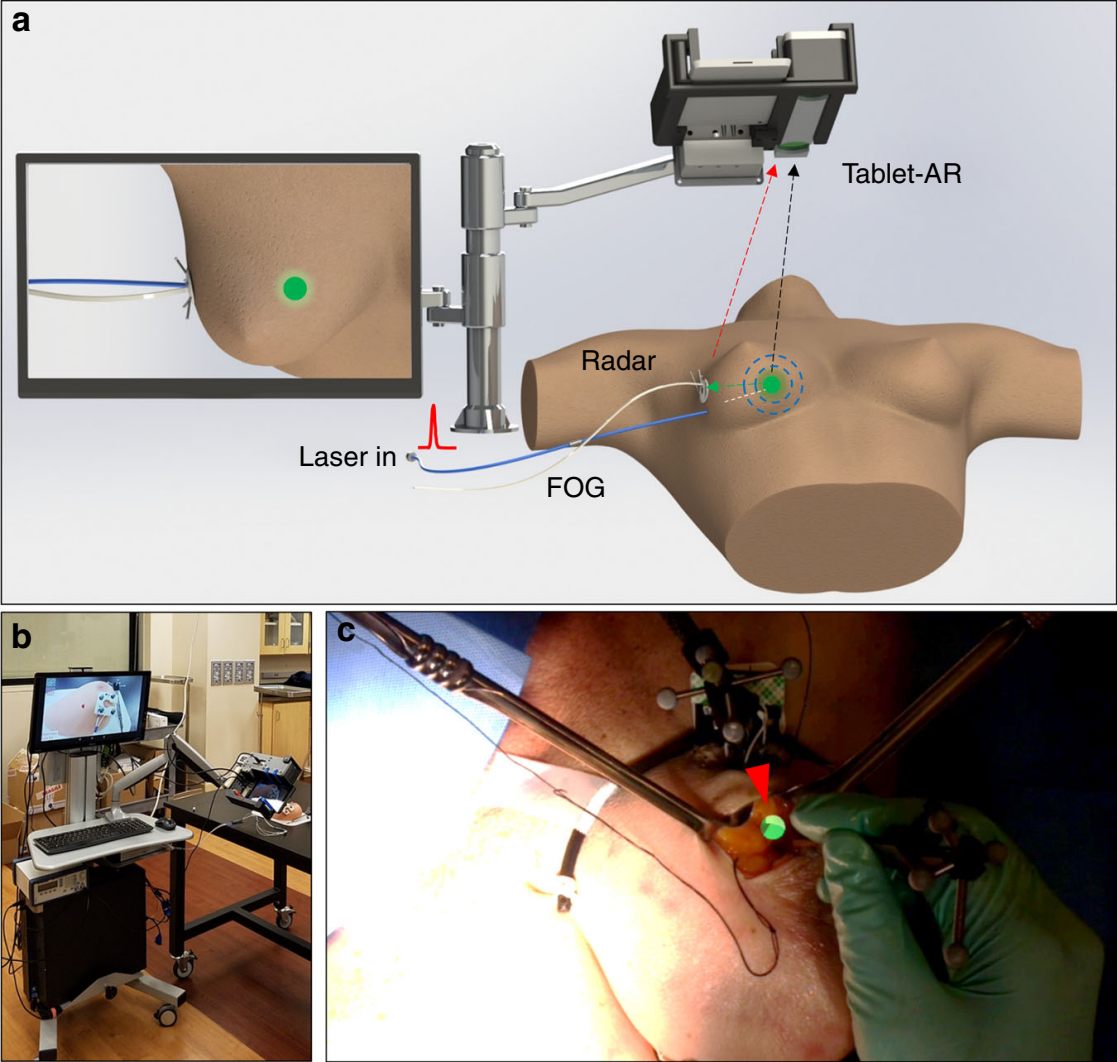
Using a fiber optoacoustic guide and an augmented reality (AR) system to locate the tumor and guide for fast and precise tumor removal. **a** Principle of using a fiber optoacoustic guide (FOG) and an AR system to locate the FOG tip and provide the visual guidance on the AR display. **b** Photograph of the compact integrated system on a cart. **c** Visualization of the FOG tip in the breast of a female human cadaver sample (green sphere and marked by a red arrow)

To address this unmet surgical need, we developed a fiber optoacoustic guide (FOG) that can locate a tumor with sub-millimeter accuracy (see Materials and methods). We further deployed an augmented reality (AR) system to transfer the obtained tumor location into an intuitive and accurate visual cue on a tablet or head-mounted display (HMD) with 0.81-mm accuracy (Fig. 1a). The FOG is a fiber-optic-based guide that is preoperatively implanted in the tumor. When connected to a pulsed laser, the FOG emits omnidirectional waves at its tip via the optoacoustic effect. Through acoustic trilateration (see Supplementary Materials) by an acoustic radar with three ultrasound transducers, the tumor can be located $(\overrightarrow {V_{F,A}} )$ with 0.25-mm accuracy. AR techniques have recently been developed for intuitive surgical navigation by merging the real-time operation with virtual information segmented from preoperative images, which then leads to improved surgical outcomes and minimal interference to the surgical flow^19–21^. Here, we leveraged the AR technology to track the acoustic radar $\left( {\overrightarrow {V_{A,AR}} } \right)$ and thus transform the obtained tumor location into an intuitive and accurate visual cue $\left( {\overrightarrow {V_{F,AR}} } \right)$ on a tablet or an HMD (see Supplementary Materials). The visual guidance requires no switch between the detection probe and the excision device, and it can guide surgeons in performing faster and more precise tumor removal. We built an AR system in both tablet and HMD forms and achieved an overall accuracy of 0.81 mm in the visualization of the FOG tip.

We further integrated the entire system into a compact transportable cart (see Supplementary Materials) for clinical validation (Fig. 1b). Using a tablet-AR system, a board-certified breast cancer surgeon successfully excised a “pseudo tumor” from the breast of a female human cadaver. Figure 1c shows a screenshot of the tablet display with the visualization of the FOG tip (green dot, marked by a red arrow) in the breast tissue of a female human cadaver in the experiments. With the intuitive and accurate visual guidance of the tumor location, our FOG, aided with the AR system, provides a tool for surgeons to perform fast and precise tumor removal. This enhanced method will have the potential to significantly reduce re-operation rates and shorten surgery time, which translates into a lower cost of care.

## Materials and methods

### Design of an FOG to locate the tumor with sub-millimeter accuracy in BCS surgery

In BCS, a surgical beacon is preoperatively implanted to mark the tumor location and guide the tumor removal in the operating room. An ideal surgical beacon in the tumor should be miniaturized (<mm diameter) to be implanted in the tumor, and it should be detectable with high accuracy over a wide range of angles and distances, while having a low cost so that it can fit into current clinical practice as a disposable device. Ultrasound, which can carry spatial information with sub-millimeter accuracy and propagates deeply into tissue, is a good candidate to serve as a localization beacon in tumors for BCS. However, the currently available single-element ultrasound transducers are either directional, that is, with limited detectable angles, or omnidirectional, which is challenging and expensive to manufacture because it consists of miniaturized spherical piezoelectric transducers that can broadcast acoustics omnidirectionally^22,23^. Moreover, spherical transducers are bulky in size and have a center frequency of several hundred kHz, which creates a wavelength of several mm and does not allow sub-millimeter localization accuracy. The fiber optoacoustic emitter, which simply attaches a thin absorption layer on the fiber tip to convert the pulsed light into acoustic waves via the optoacoustic effect, has become an emerging acoustic source because of its broadband acoustic emission bandwidth and ease of fabrication^24,25^. However, the current fiber optoacoustic emitters focus on generating directional acoustic emission for conventional ultrasound imaging^24^, which is not a good fit in BCS for the wide detectable angular range that is needed. Additionally, simply attaching the absorption layer at the fiber tip is prone to breaking because of the tight confinement of the high laser energy at the fiber tip.

**Fig. 2:**
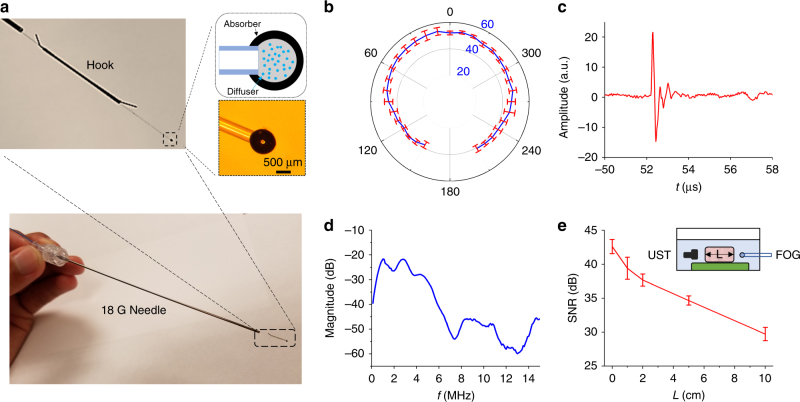
A fiber optoacoustic guide for tumor localization with sub-millimeter accuracy. **a** Photograph of the fiber optoacoustic guide (FOG), zinc oxide nanoparticles, and epoxy forming the diffuser layer to diffuse the light, and the graphite and epoxy layer converts the light into an optoacoustic signal. **b** Signal-to-noise ratio (SNR) of the generated optoacoustic signal from the FOG tip at different angles. The radius of the data points marks its SNR (in dB): the further the data point lies, the higher the SNR is. **c** Representative optoacoustic signal waveform recorded 8 cm away from the FOG tip in the forward direction. **d** Frequency spectrum of the representative optoacoustic signal waveform after normalization of the detector’s response. **e** SNR of the generated optoacoustic signal after passing chicken breast tissue of different thicknesses: 0, 1, 2, 5, and 10 cm. The inset shows the measurement setup

Here, we developed a two-layer nano-composite structure at the fiber tip to generate acoustic waves in all directions with sufficient intensity to penetrate deeply in the tissue. The first layer comprises 100-nm zinc oxide (ZnO) nanoparticles and epoxy (15% concentration by weight), which diffuses the high-energy laser pulse into a relative uniform angular distribution (Fig. S1). The second layer is an absorption layer of graphite and epoxy (30% concentration by weight), which completely absorbs the diffused laser pulse (Fig. S2) and transforms it into omnidirectional acoustic waves. The nano-composite layer was coated on the polished tip of one multimode optical fiber (200 µm core diameter, FT200EMT, Thorlabs, Inc., Newton, NJ, USA) by two repetitive dipping steps (see Supplementary Material). The finalized ball-shaped nano-composite layer has an overall diameter of approximately 800 µm (Fig. 2a). This spherical geometry at close to a millimeter size also facilitates the generation of acoustic waves with frequencies of several MHz, which penetrate deeply into the breast tissue and provide a long detectable distance. Last, a hook sleeve is attached at the distal end to prevent migration in the tissue. The finalized FOG is threaded into an 18 G positioning needle used for the current metal guide wire, minimizing the translational cost.

### Measuring the acoustic emission profile and the frequency of the FOG

A customized and compact passively Q-switched diode-pumped solid state laser (1030 nm, 3 ns, 100 μJ, RPMC, Fallon, MO, USA) was used as the excitation source for the FOG. The laser is a fiber pigtailed laser, and it was connected to the FOG through a homemade fiber jumper (SMA-to-SC/PC, ~81% coupling efficiency). The laser driver was adjusted to set the output power from the fiber jumper to be approximately 34 mW, which corresponds to a pulse repetition rate of approximately 420 Hz. The FOG tip was fixed in the water tank. One miniaturized ultrasound transducer (XMS-310-B, Olympus, Waltham, MA, USA) was mounted on a motorized rotation stage to record the optoacoustic signals across different angles. An additional three-dimensional (3D) translation stage was used to adjust the FOG to make its tip well-centered. The distance from the FOG tip to the transducer was 80 mm. The ultrasonic signal was first amplified by an ultrasonic pre-amplifier (0.2–40 MHz, 40 dB gain, Model 5678, Olympus, Waltham, MA, USA) and then sent out to an oscilloscope (DSO6014A, Agilent Technologies, Santa Clara, CA, USA) to read out. The signal was averaged 16 times. By rotating the motorized stage, the peak-to-peak values and the waveforms of the optoacoustic signal were recorded at different angles, and the signal-to-noise ratios (SNR) were calculated. All of the devices were synchronized by the output from the active monitoring photodiode inside the laser.

### Measuring the penetration depth of the acoustic signal generated by the FOG

The inset in Fig. 2e shows the experimental schematic used to investigate the penetration depth of the optoacoustic signal that was generated. The FOG and the same miniaturized ultrasound transducer were submerged in a water tank and separated by approximately 13 cm. The miniaturized ultrasound transducer was mounted on a three-axis translation stage. The same laser was applied as the excitation source in this setup with the same power. First, no chicken breast tissue was placed between the ultrasound transducer and the FOG. We aligned the ultrasound transducer with the FOG by adjusting the three-axis translation stage to obtain the optimal optoacoustic signal. Then, chicken breast tissues of different thicknesses were placed between the ultrasound transducer and the FOG. The detected signal peak-to-peak values were then recorded using a similar setup as above, and the SNRs were calculated.

### Characterizing the acoustic/optical tracking accuracy of the FOG and AR system

#### Acoustic tracking accuracy of the FOG tip

The FOG was mounted on a 3D manual translation stage. The acoustic radar, which has three identical miniaturized transducers (XMS-310-B, Olympus, Waltham, MA, USA), is fixed on a post. Both the FOG and the acoustic radar were submerged in a water tank. The three transducers simultaneously acquired the acoustic signal generated by the FOG tip. The signals were amplified by three identical pre-amplifiers (40 dB gain) and then sent to a host PC with an integrated data-acquisition card (Oscar 16, 50 MS/s, DynamicSignals, Lockport, IL, USA). Through processing the delay of the recorded acoustic signal to the excitation pulse, the distances of the FOG tip to each transducer were obtained and then used to calculate the 3D position of the FOG tip relative to the acoustic radar via a trilateration algorithm. We shifted the FOG with a given physical shift of 0.05 inches each time using the 3D manual stage along its *x*, *y*, and *z* axes and recorded the calculated 3D positions by the trilateration algorithm. By comparing the calculated shifts against the physical shifts by the stage along *x*, *y*, and *z* axes, the mean errors of the trilateration for the *x*, *y*, and *z* movements were characterized.

#### Optical tracking accuracy of the acoustic radar by the tablet-AR system

The acoustic radar that tracks the FOG tip in tissue is tracked through infrared (IR) markers by a stereo camera in the tablet-AR system for later AR visual cue projection. The IR marker group on the acoustic radar was fixed on a 3D manual translation stage. The stereo vision system was placed ~40 cm away from the marker group, which was approximately the same distance as the setup used in the cadaver experiments. The stereo vision system was kept static during the whole process. The marker group was shifted at a fixed physical step of 0.05 inches each time on the 3D manual stage along its *x*, *y*, and *z* axes. At each step, the position of the rigid body’s pivot point, that is, the center of all markers in the group, was streamed to the hosting PC in real time and recorded. The marker group movement was calculated from the optical tracking results and compared against the physical shift of the manual stage along the *x*, *y*, and *z* axes.

#### Projection accuracy of the FOG tip on the tablet-AR system

A square marker mount with a 4 × 4 checkerboard was used to measure the projection error of the tablet-AR system used in the cadaver experiments. The four markers were mounted on the 3D-printed square board and formed a rigid body that is coplanar and concentric with the checkerboard. The center of the rigid body served as a pseudo “FOG tip” position, where a sphere cue is rendered. The tablet-AR system was placed at ~40, ~50, and ~60 cm from the checkerboard. At each distance, multiple screenshots that contain both the rendered sphere cue and the checkerboard were taken at various heights and orientations. For each screenshot, the 2D position of the visual cue rendered on the tablet display was obtained from a robust circle detection method, while the 16 checkerboard corners were detected using the method used in camera calibration (see Supplementary Material), and their averaged center served as the ground truth of the rendering. The absolute error between the rendered pseudo “FOG tip” position and the ground truth provided by the checkerboard was measured in pixels at each distance. With the physical distance and pixel distance between the two corners farthest away on the checkerboard, the pixel-to-mm ratio on each screenshot was later obtained to transfer the error from pixels to millimeters.

### Female human cadaver sample

The female human cadaver sample was secured through the surgery skill lab of the Department of Surgery, Indiana University School of Medicine, as a surgery training sample. The female human cadaver sample is a female of 66 years old and was deceased from a malignant brain tumor. The experiment protocol was reviewed and determined to be Internal Review Board exempt through the determination form of exempt research request by the human research protection program of Purdue University: https://www.irb.purdue.edu/forms/. The entire experiment was supervised by the surgery skill lab at the Indiana University School of Medicine, and the whole cadaver sample was collected and cremated according to standard practice by The Cremation Center (1601 East New York Street, Indianapolis, IN, USA) after the experiment.

### Implantation of a breast biopsy clip to mark a “pseudo tumor” for excision

Since a female human cadaver with a breast tumor is difficult to obtain, we instead placed a standard breast biopsy clip to represent the core of the “pseudo tumor,” which was to be excised in our experiment. The breast tumor biopsy clip is a small metal (stainless steel or titanium) clip that is inserted into the breast to mark the biopsy site, and it has been used as a guide to locate and remove nonpalpable tumors^26^. With the imaging guidance of a portable ultrasound imaging system (*Lumify* ultrasound, C5-2 probe, Philips), a board-certified radiologist identified a target position at approximately 10–20 mm depth in breast tissue with compression applied. Then, the radiologist percutaneously deployed a biopsy clip through a needle (*UltraClip* breast tissue marker, Bard, Murray Hill, NJ, USA) into the position and released the biopsy clip. After the release of the biopsy clip, an ultrasound image sequence and snap shots were recorded to confirm the placement of the biopsy clip in the breast tissue.

### Surgical procedure for removing a “pseudo tumor” from a female human cadaver

#### Step 1: Implantation of the FOG

After the deployment of the biopsy clip, the ultrasound probe was held in the same position, and the radiologist percutaneously placed the two-part positioning needle with a trocar introducer (G19380, Kopans Breast Lesion Localization Needle, Cook Medical, Bloomington, IN, USA) into a position close to the implanted biopsy clip. The two-part positioning needle was inserted into the breast tissue along the direction from the medial to the lateral side. Because the visualization of the needle sometimes might not be clear in ultrasound imaging, the radiologist gently pushed and pulled the needle to record an ultrasound image sequence to confirm that the location of the needle was close to the implanted biopsy clip. The core needle was removed, and the introducer was left inside the tissue. The same loading cannula that comes with the introducer and had our optoacoustic guide wire encapsulated was then fed into the introducer in the tissue. Next, the radiologist advanced the FOG, released the hook, and then removed the needle and the introducer. Then, ultrasound images and sequences were taken to confirm the placement of the FOG and its tip’s close distance to the implanted biopsy clip. The guide wire outside the breast tissue was later taped to the skin for the subsequent visualization and excision experiments.

#### Step 2: Visualization of the FOG tip in the breast tissue with the surgical navigation system

With the implantation of the biopsy clip and FOG, our surgical navigation system was connected and turned on to visualize the location of the FOG tip in the human breast tissue. First, the proximal end of the implanted FOG was connected to the compact pulsed laser. Second, the acoustic radar was patched to the breast skin close to the insertion site of the FOG by having its base attached to the medial skin through a sticky pad. The supporting arm of the acoustic radar was adjusted to ensure that the acoustic radar was in firm contact with the breast skin. Next, the tablet-based AR system mounted on the articulating arm was moved to a position above the breast to capture the entire operating scene and the acoustic radar. Then, the host PC was turned on to receive and process the acoustic signals. The tablet-AR system was started later to receive the tracking information from the host PC and render the visualization of the FOG tip on its display. The tablet display was also streamed to a secondary monitor that is easier and friendlier for operators to view. As a result, the FOG tip in the breast tissue was visualized on the monitor. The tablet-AR system can be moved to different positions to obtain a better view at the operators’ preference.

#### Step 3: Excision of a “pseudo tumor” from a female human cadaver with visualization guidance by the surgical navigation system

In addition to the visualization of the location of the FOG tip in the breast tissue, our system also provided the real-time distance from the tip of the scalpel to the FOG tip to enable accurate tissue removal. In addition, an auditory warning was given when the scalpel tip’s distance to the FOG tip was less than the preset warning distance. In the excision experiment on the cadaver, the warning distance was set to 25 mm. A board-certified breast surgeon successfully performed excision of the “pseudo tumor” marked by the biopsy clip following the protocol showed later in the results section.

## Results and discussion

### FOG omnidirectionally broadcasts the acoustic signal deep in the tissue

Figure 2a shows that the FOG is a fiber-optics-based guide with a nano-composite formed at its distal end, which has an overall diameter of approximately 800 μm. Under external laser excitation of ~0.1 mJ pulse energy, at a 420-Hz repetition rate, the FOG successfully broadcasts an acoustic signal with more than a 40-dB SNR (averaged 16 times, ~25-Hz final data refresh rate) over a 300° angular range in water (Fig. 2b, see Supplementary Material). This angular range result is limited by the rotatable angles of our setup. A backward acoustic emission was observed in both water and human breast tissue, which proves an omnidirectional acoustic emission of FOG. Figure 2c shows a representative optoacoustic signal measured in the experiment. Its radiofrequency spectrum spans mainly the low-frequency range of 1–5 MHz (Fig. 2d) after normalization of the transducer response. Since the frequency of an optoacoustic emitter relates to its geometry^27^, the diameter of the composite sphere being close to 1 mm could be one of the reasons for such a low radiofrequency spectrum. Compared with the high-frequency ultrasound wave, the generated optoacoustic wave can penetrate much deeper through human tissue due to its low-frequency nature. An SNR as high as 30 dB after passing through the 10-cm-thick chicken breast tissue was obtained in our experiments (Fig. 2e). This long detection distance of the FOG fits well in the current BCS operations, in which the guide wire is approximately 5–10 cm long. We note that laser diodes with a longer pulse duration could be used as an alternative excitation light source to lower the frequency of the optoacoustic signal^28^, which also reduces the cost of the system.

### FOG locates the tumor with sub-millimeter accuracy

**Fig. 3:**
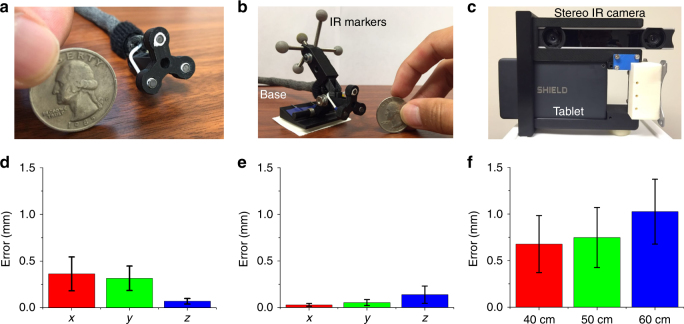
Accuracy of the ultrasonic and optical localization and rendering on the AR display. **a** Photo of the acoustic radar with three transducers. **b** Photo of the acoustic radar with infrared (IR) markers and the mount base. **c** Photo of the tablet-based AR system. **d** Tracking accuracy of the FOG tip in water by the acoustic trilateration when it moves on the *x*, *y* (lateral directions), and *z* (axial direction) axes, respectively. **e** Optical tracking accuracy of the acoustic radar through a stereo IR camera when the radar moves on the *x*, *y* (lateral directions), and *z* (axial direction) axes, respectively. **f** Projection accuracy of a given target point on the AR display when it has movement on the *x*, *y*, and *z* axes at ~40, ~50, and ~60 cm distances, respectively

We fixed the acoustic radar with three transducers (Fig. 3a.

) to detect the acoustic signals to locate the FOG tip and calculate the measured shifts. Compared with the physical shifts applied, the mean error of the acoustic tracking is 0.36, 0.32, and 0.10 mm for movement on the *x*, *y*, and *z* axes, respectively, and the overall mean error is 0.25 mm (Fig. 3d). The above specifications demonstrate a low-cost, compact, and high-intensity omnidirectional acoustic emitter that is suitable for use as an “acoustic beacon” for tumor localization.

**Fig. 4:**
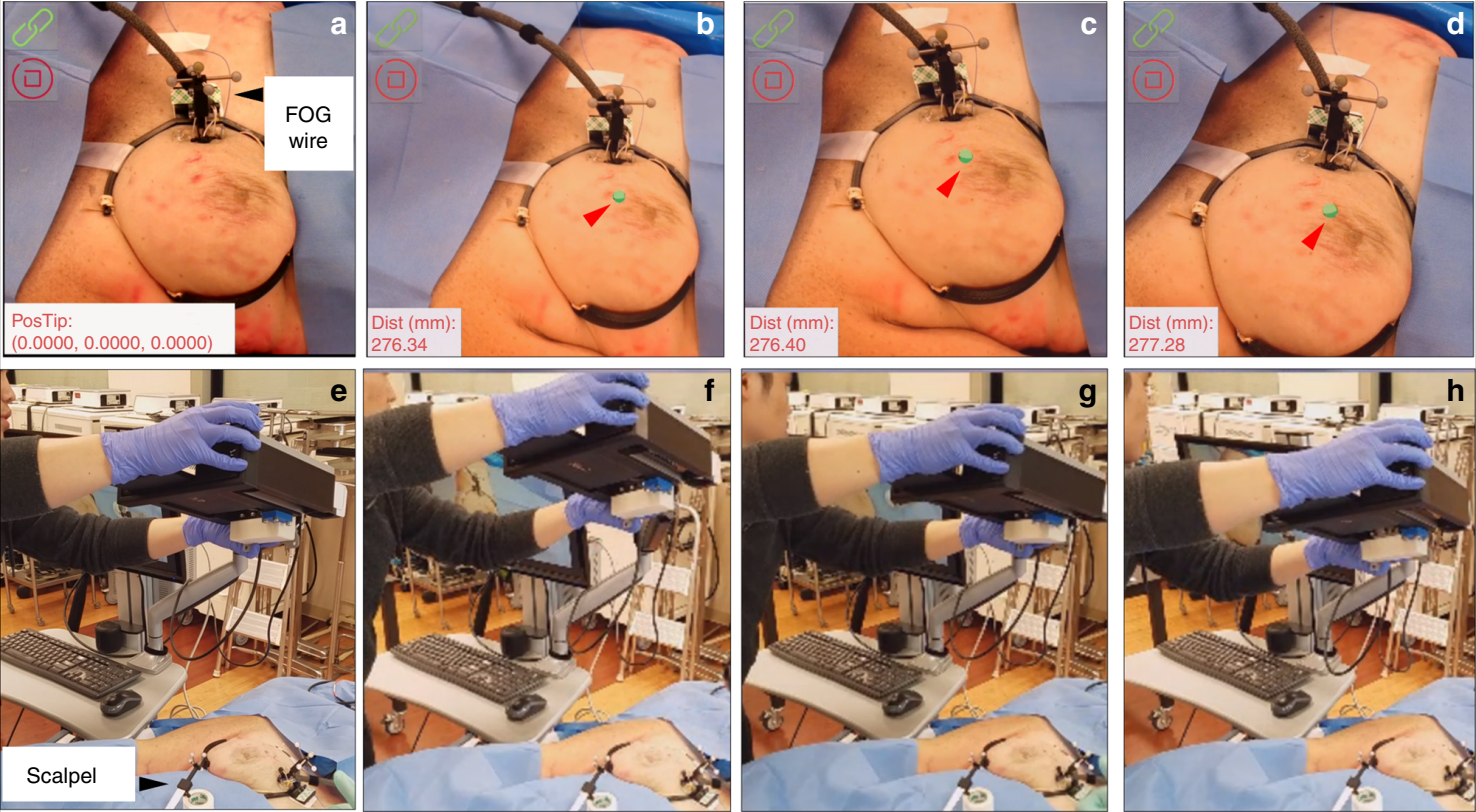
Visualization of the FOG tip in the breast tissue of a female human cadaver sample using the tablet-AR system at different view angles and distances. **a** It is difficult to estimate the location of the FOG tip in the breast from the guide wire outside the breast (black arrow) before turning on our system. **b**–**d** Clear visualizations of the FOG tip in the tissue on the AR display at different view angles and distances. The real-time distance of the static scalpel tip to the FOG tip is displayed on the bottom left of the AR display. **e**–**h** The positions of the tablet-AR system relative to the breast tissue in **a**–**d**, respectively

### AR for real-time tumor localization with millimeter-level accuracy

By placing the IR markers on the acoustic radar (Fig. 3b) and using a similar setup, we translated the acoustic radar with given physical shifts along the *x*, *y*, and *z* axes and compared it with the calculated shifts by optical tracking using a tablet-AR system (see Supplementary Material) with a stereo IR camera (Fig. 3c). The mean error of the depth sensing of the acoustic radar was measured to be 0.03, 0.05, and 0.14 mm for the *x*, *y*, and *z* movement, respectively, and the overall mean error was 0.07 mm (Fig. 3e).

Next, we used a customized checkboard with IR markers to compare the projected visual cue through our calculation against the ground truth obtained from the captured image of the checkboard. The error of projecting the calculated position of the FOG tip on the tablet display was assessed. The tablet-AR system was placed at approximately 40, 50, and 60 cm away from the checkboard in the experiments to measure the projection error, which is approximately the distance of the AR system to the operating scene in practice. It is observed that the mean projection errors are 0.68, 0.75, and 1.03 mm at the distances of 40, 50, and 60 cm, respectively, and the overall mean projection is 0.81 mm (Fig. 3f).

In clinical practice, another important factor that would contribute to the localization error is the sound speed difference caused by the tissue inhomogeneity. Acoustic waves travel faster in malignant tissue. The sound speeds in malignant tissue (mostly palpable) and normal tissue are ~1552 and ~1472 mm/s, respectively, in the most extreme case^29^. This sound speed difference is caused by the change in the mechanical property. In our application, we focus on the nonpalpable breast tumor, which has a median size of approximately 13.8 mm and a sound speed closer to the normal tissue than the palpable tumors^30^. Thus, the localization error caused by tissue inhomogeneity is estimated to be <0.4 mm in the most extreme case described. This tissue inhomogeneity error is still within the sub-millimeter level, similar to in the acoustic, optical tracking, and projection errors of our system.

By characterizing the error of the acoustic localization, optical tracking, projection, and tissue inhomogeneity, it is found that the projection error is the largest error, but all of the four errors are at a sub-millimeter level, which suggests an overall millimeter-level accuracy of our system. Therefore, our FOG together with the tablet-AR system demonstrates an intuitive visual guidance of the tumor location with a millimeter-level accuracy, which fulfills the need for accurate tumor localization.

### Visualization of the FOG tip in breast tissue in a female human cadaver

With the implantation of the FOG in the left breast of a female human cadaver, the tablet-AR system was set up to visualize the FOG tip in the breast. Before turning on the system, through the guide wire outside the breast (marked by the black arrow in Fig. 4a), it was difficult to estimate the location of the FOG tip in the breast. After turning on the system, the FOG tip’s location in the breast was clearly visualized on the tablet display, which is represented by green dots and marked by red arrows in Fig. 4b–d. The FOG tip was always visualized (Fig. 4b–d), even when the tablet was placed at different heights and angles to the breast (Fig. 4f–h). Additionally, by attaching another group of IR markers to the surgical scalpel, the real-time distance of the scalpel tip to the guide wire tip was obtained as additional guidance (see Supplementary Material). Through reading this real-time distance on the bottom left of the tablet display over Fig. 4b–d, the visualization was proved to be quite stable. The distances were calculated to be 276.67 mm on average, with a maximum deviation of 0.61 mm, which is approximately the same level of overall tracking accuracy of our system. The stable visualization of the FOG tip can also be seen from the Movie S1, in which the visualization of the FOG tip was recorded when the tablet was in motion, and a 50-time signal average was used in the cadaver experiments.

**Fig. 5:**
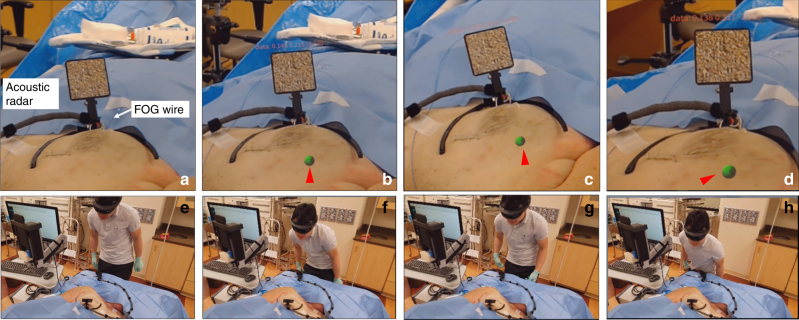
Visualization of the FOG tip in the breast tissue of a female human cadaver sample using the head-mounted display AR system (Hololens) over different view angles and distances. **a** It is difficult to estimate the location of the FOG tip in the breast from the guide wire outside the breast (white arrow) before turning on the system. An image target is used instead of IR markers to track the acoustic radar. **b–d** Clear visualizations of the FOG tip in the tissue on the stereo view from the head-mounted display (HMD) on the operator’s head when the operator views the operating scene at different view angles and distances. **e**–**h** The positions of the HMD-AR system relative to the breast tissue in **a–d**, respectively

To further reduce the surgical interference, we also explored the use of an HMD-AR system (Hololens, Microsoft, Syracuse, NY, USA) to visualize the FOG tip in the breast tissue in the first-person view of the operator. As a proof-of-concept, we used the RGB camera built in the Hololens to sense the acoustic radar. Thus, instead of using passive IR markers, an image target was placed on the acoustic radar for the RGB camera tracking (Fig. 5a) (see Supplementary Material). In view of the operation captured by the Hololens camera, it was difficult to estimate the location of the FOG tip in the breast tissue before turning on the system (Fig. 5a). After turning on the system, the FOG tip’s location in the breast was clearly visualized on the Hololens display, which is represented by green spheres and marked by red arrows in Fig. 5b–d. When viewing the operating scene over different distances and angles (Fig. 5f–h) to the breast, the visualization of the FOG tip in the breast was stable (Fig. 5b–d). This is clear in the recorded video when the operator moved with the Hololens (Movie S2). Thus, using an HMD-AR system, visual guidance in the first-person-view of the operator can be provided, which has the potential to achieve hands-free surgical guidance with the least interference to the flow.

**Fig. 6:**
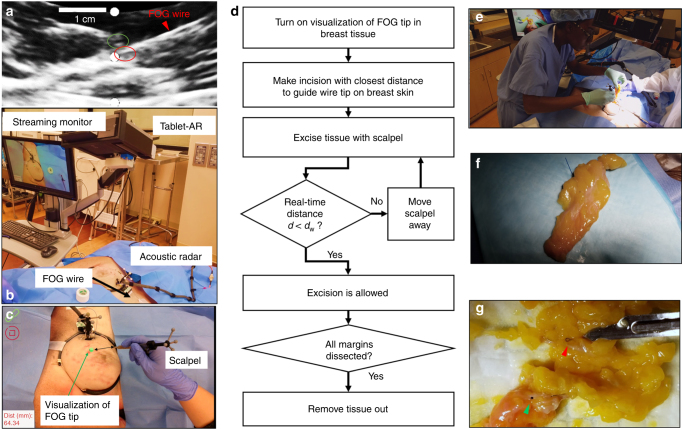
Excision of a “pseudo tumor” marker by a biopsy clip in the breast of a female human cadaver using the fiber optoacoustic guide and the tablet-AR system. **a** Ultrasound image that confirms the implantation of the guide wire and biopsy clip in the tissue. The FOG tip (red circle) is close to the implanted biopsy clip (green circle). **b** Setup of the system in the excision experiment. **c** Visualization of the FOG tip in the breast tissue (green dot) and the real-time distance of the scalpel tip to the FOG tip on the AR display. **d** Protocol of the excision of the “pseudo tumor” in the experiments. **e** Photo of the board-certified breast cancer surgeon using the system to excise the tumor. **f** Photo of the excised “pseudo tumor”. **g** Photo of the cut-open excised tissue, in which the implanted biopsy clip and the FOG tip are inside, which represents a successful excision of the “pseudo tumor”. The green arrow shows the FOG tip, and the red arrow shows the biopsy clip

Compared to a conventional AR surgical guidance system, our FOG-AR system locates the target lesion in soft tissue with higher accuracy and therefore offers more precise surgical guidance because a conventional AR system utilizes the target lesion information segmented from static preoperative images, and soft tissue is subject to a large amount of movement and deformation during surgery. In contrast, our FOG implanted in the tumor moves along with the tumor when tissue movement and deformation occur. The FOG dynamically locates the target lesion during the surgery, and the AR system converts it into an intuitive visual guidance in real time. Therefore, our technology has higher resistance to the tissue movement and deformation and thus locates the target lesion in soft tissue with higher accuracy.

### Excision of a “pseudo tumor” in a human cadaver guided by FOG and tablet-AR

To validate the efficacy of the FOG and the tablet-AR system, we performed an excision experiment of a “pseudo tumor” marked by a pre-implanted biopsy clip in the breast of a female human cadaver. In the experiments, the FOG was placed close to the biopsy clip as the guide for visualization of the tumor location by a board-certified radiologist. Both the biopsy clip and the FOG were implanted in the breast by a board-certified radiologist with specialty training in breast imaging through a standard clinical practice method (Movie S3). The locations of the biopsy clip and the FOG tip were confirmed to be at a close distance in the ultrasound image (Fig. 6a) and Movie S3. After the implantation, the tablet-AR system was set up (Fig. 6b). Figure 6c shows the visualization of the FOG tip on the tablet display in the experiment. By mounting IR markers on the off-shelf scalpel used and tracking the scalpel with those markers, the real-time distance from the scalpel tip to the FOG tip was obtained and displayed on the tablet display as additional guidance.

By following the excision protocol (Fig. 6d), a board-certified breast cancer surgeon performed the excision experiment. After turning on the system and visualizing the FOG tip in the breast, the surgeon first made the incision on the skin close to the FOG tip. Then, the surgeon excised the pseudo tumor with a scalpel. An auditory alert was provided to the surgeon when the real-time distance of the scalpel tip to the FOG tip was less than the preset warning distance *d*_w_. The warning distance was set to be 25 mm in the experiment. In contrast to methods using a handheld probe to locate the tumor, our visualization guidance is more intuitive, and the auditory feedback on the scalpel’s distance to the tumor requires no switch between the excision device and the detection probe, which greatly reduces the interference to the surgical flow. Figure 6e shows the image of the surgeon using the scalpel and our system to excise, and the complete excision process was recorded in Movie S4. After dissecting all of the margins and cutting the guide wire, the “pseudo tumor” was excised (Fig. 6f). Due to the low contrast of the low-frequency portable ultrasound imaging probe, we transected the excised tissue to confirm that both the FOG tip and the biopsy clip (Fig. 6g) were included in the resection.

## Conclusions

We developed a compact, mobile surgical navigation system that utilizes a FOG to locate a tumor with 0.25-mm accuracy and an AR system display on a tablet to visualize the tumor location with a 0.81-mm mean error at a 25-Hz data refresh rate. The FOG dynamically locates the target lesion during surgery, and the AR systems convert it into an intuitive visual guidance in real time. Therefore, our system has higher resistance to the tissue movement and deformation and thus locates the target lesion in soft tissue with higher accuracy. To improve the excision efficiency and accuracy, our system can be further applied for accurate target lesion localization and removal in other soft tissues, such as kidney and liver, which demand precise lesion removal and maximum normal tissue preservation^31,32^. Towards clinical translation, a surgeon successfully deployed the FOG to excise a “pseudo tumor” in a female human cadaver. By offering sub-millimeter tumor localization and intuitive real-time surgical guidance, our system can help to achieve precise and fast tumor removal, reduce the high re-operation rates and therefore reduce the cost of care. Moreover, the real-time localization of the target lesion by our system with a sub-millimeter accuracy could be exploited to achieve precise supervised/semi-automatic or automatic surgeries, such as laparoscopic surgeries, in soft tissue when combined with surgery robots^33^.

### Data and materials availability

The authors declare that all of the data supporting the findings of this study are available within the paper and the supplementary information.

## Electronic supplementary material

Supplementary Figure s5(TIF 2316 kb)

Supplementary material(DOCX 3275 kb)

Supplementary Figure s1(TIF 693 kb)

Supplementary Figure s2(TIF 1819 kb)

Supplementary Figure s3(TIF 722 kb)

Supplementary Figure s4(TIF 6536 kb)

Supplementary Figure s6(TIF 5038 kb)

Supplementary Movie S1

Supplementary Movie S2

Supplementary Movie S3

Supplementary Movie S4
