# Supplementary figures and images for: A fiber optoacoustic guide with augmented reality for precision breast-conserving surgery
Source: Light Sci Appl. 2018 May 18;7:2. doi: 10.1038/s41377-018-0006-0 (PMC6107008; doi:10.1038/s41377-018-0006-0)

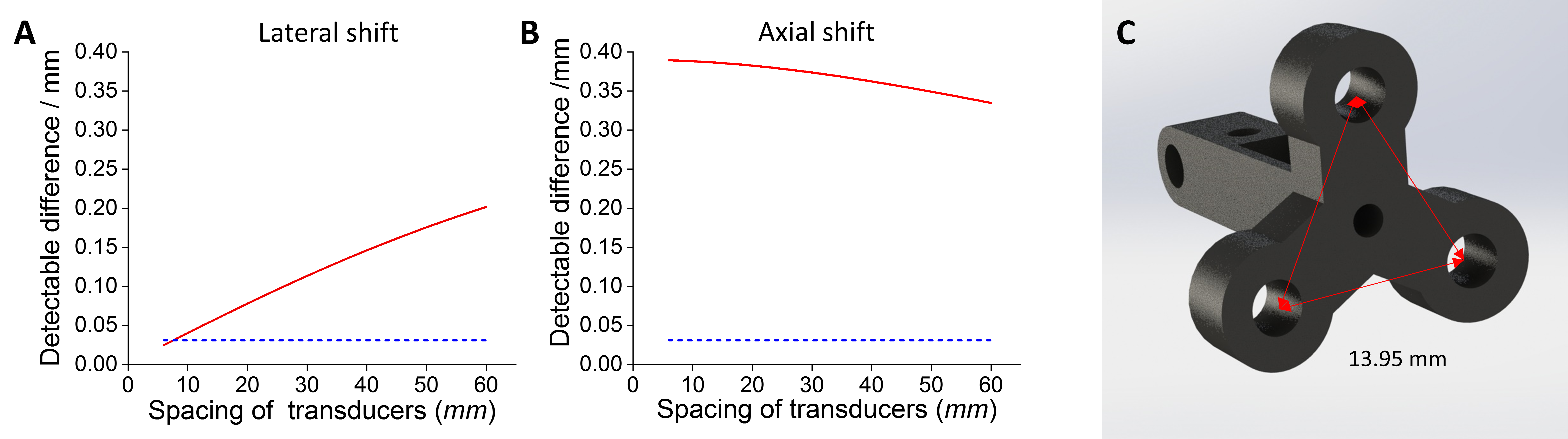

Supplement: Supplementary file 1 — Supplementary Figure s5(TIF 2316 kb) [file 41377_2018_6_MOESM1_ESM.tif]

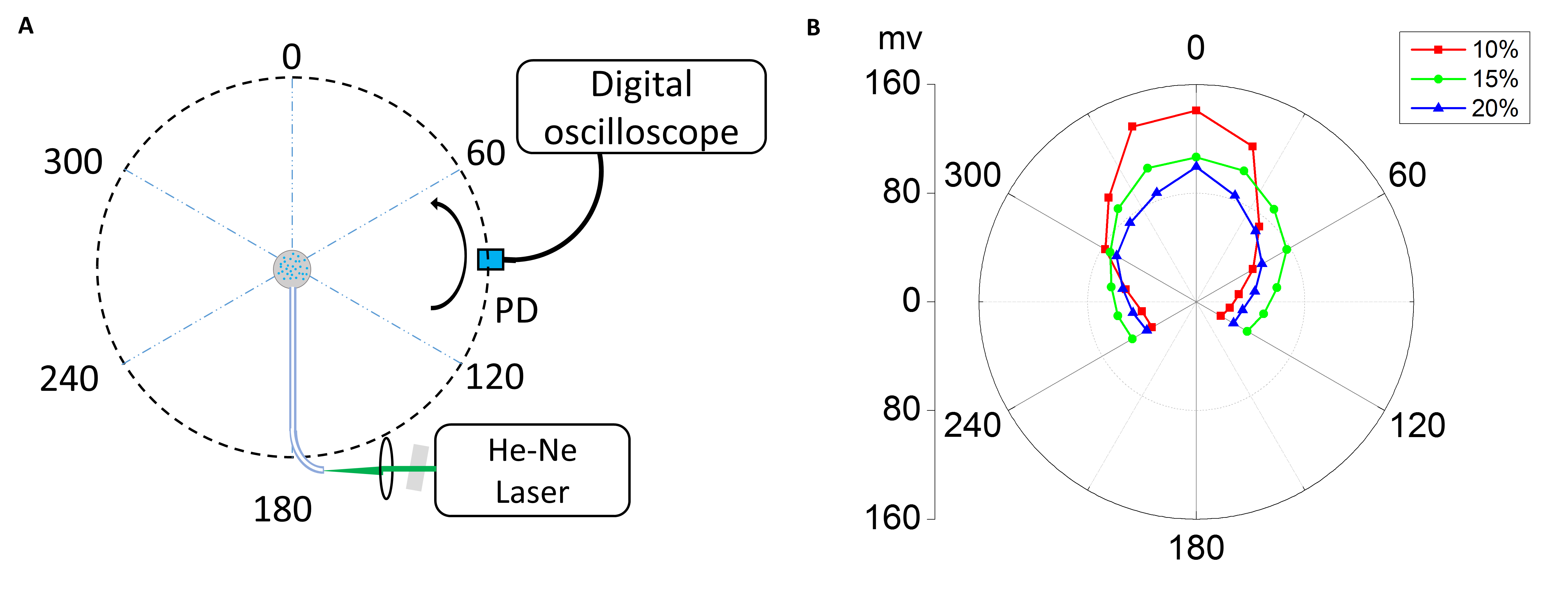

Supplement: Supplementary file 3 — Supplementary Figure s1(TIF 693 kb) [file 41377_2018_6_MOESM3_ESM.tif]

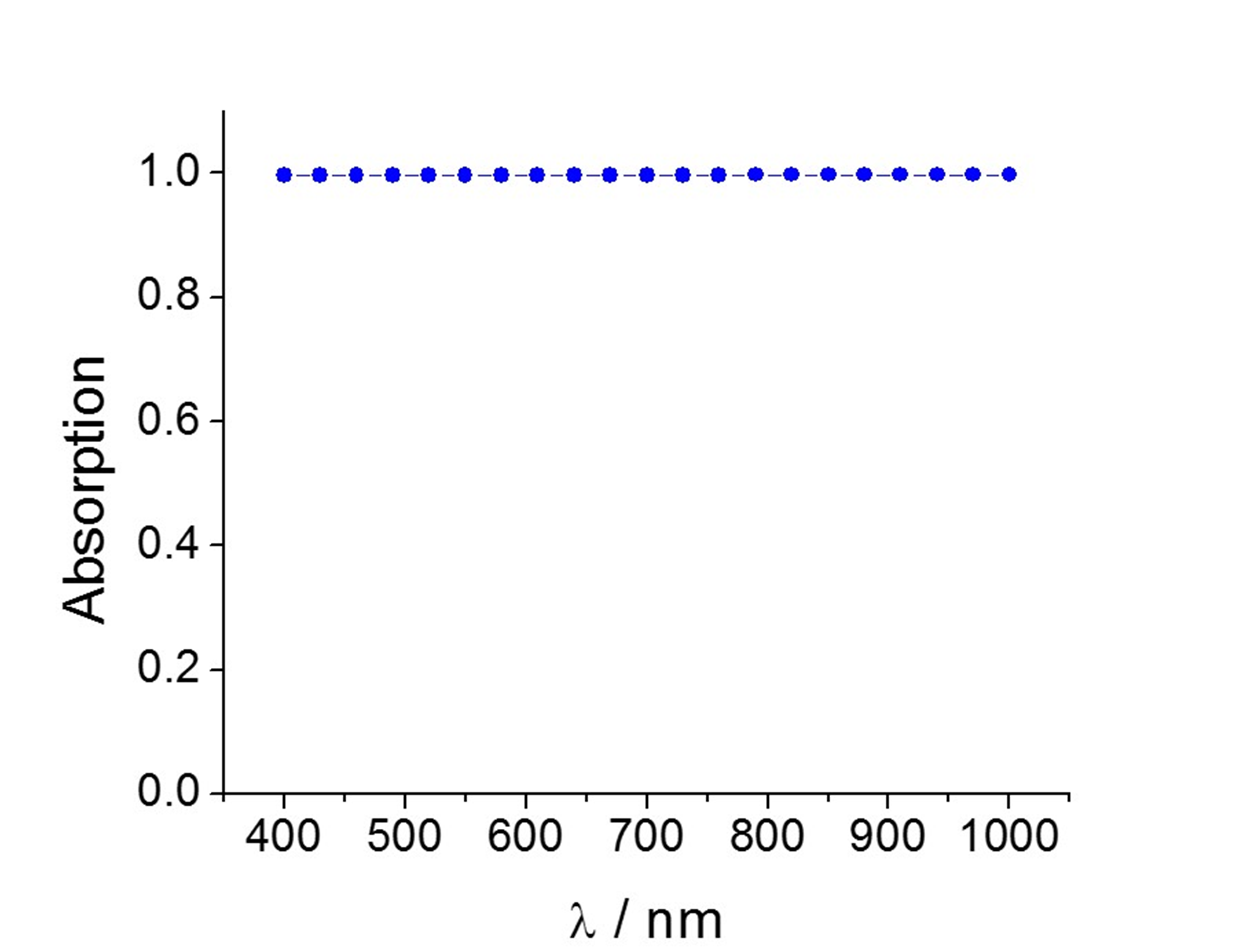

Supplement: Supplementary file 4 — Supplementary Figure s2(TIF 1819 kb) [file 41377_2018_6_MOESM4_ESM.tif]

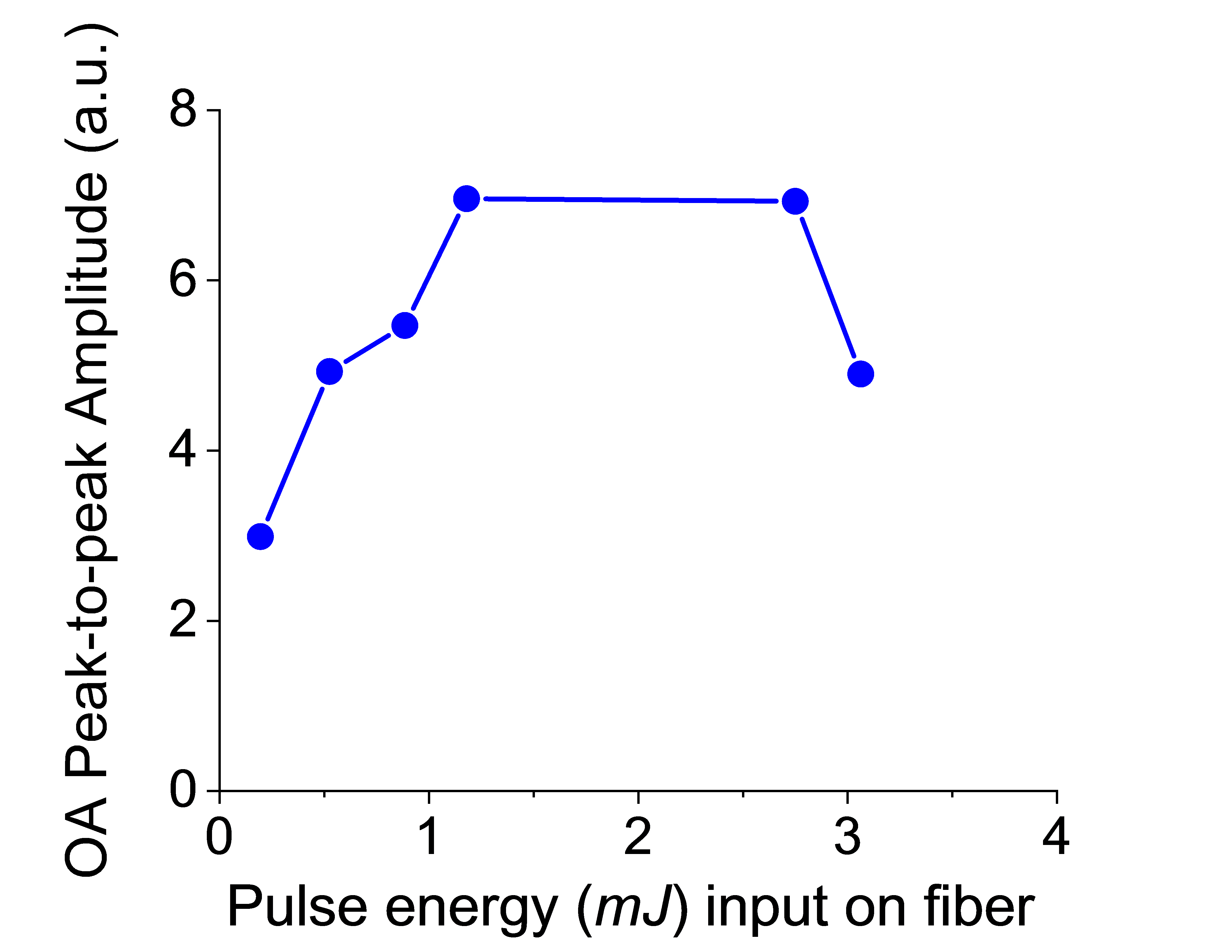

Supplement: Supplementary file 5 — Supplementary Figure s3(TIF 722 kb) [file 41377_2018_6_MOESM5_ESM.tif]

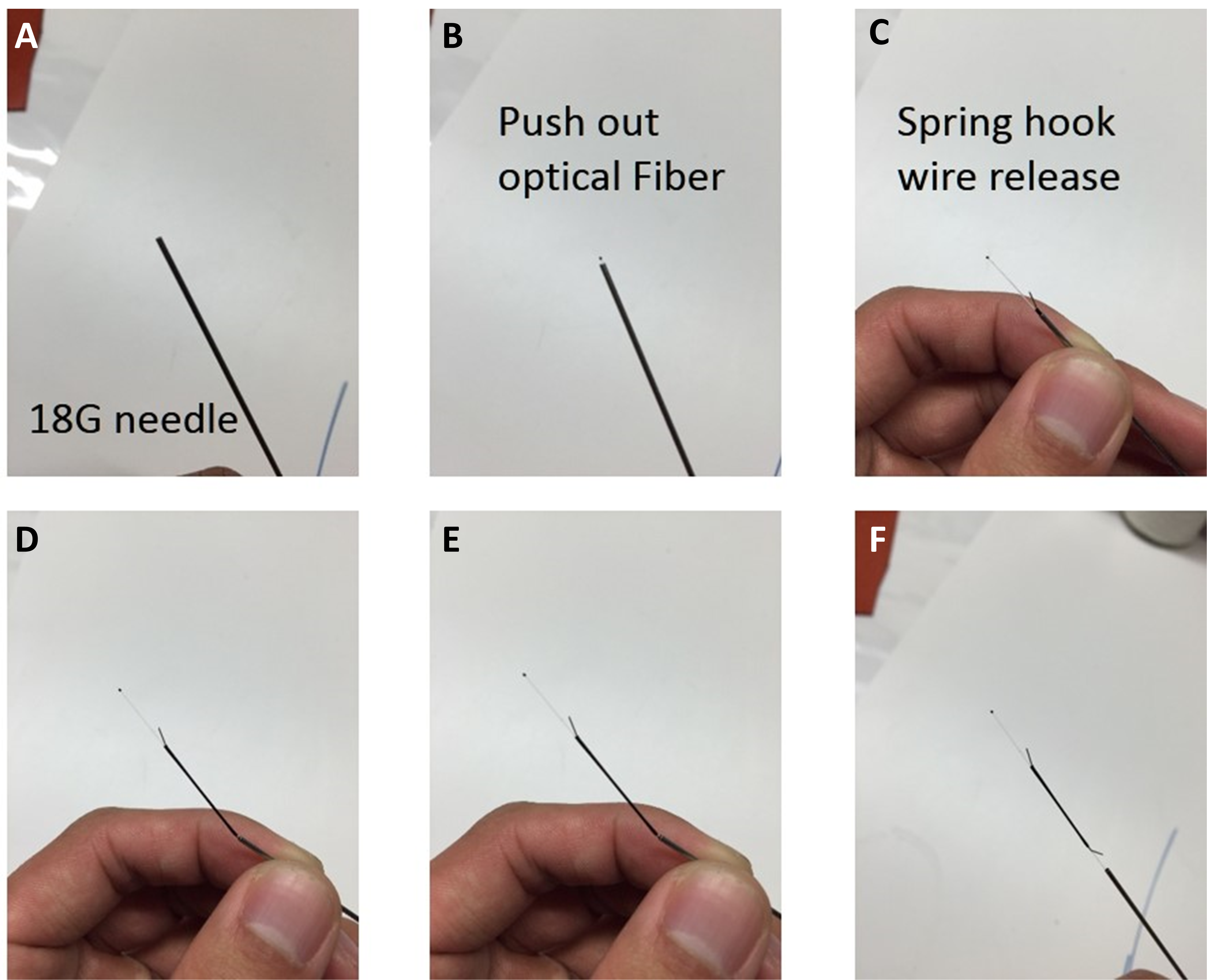

Supplement: Supplementary file 6 — Supplementary Figure s4(TIF 6536 kb) [file 41377_2018_6_MOESM6_ESM.tif]

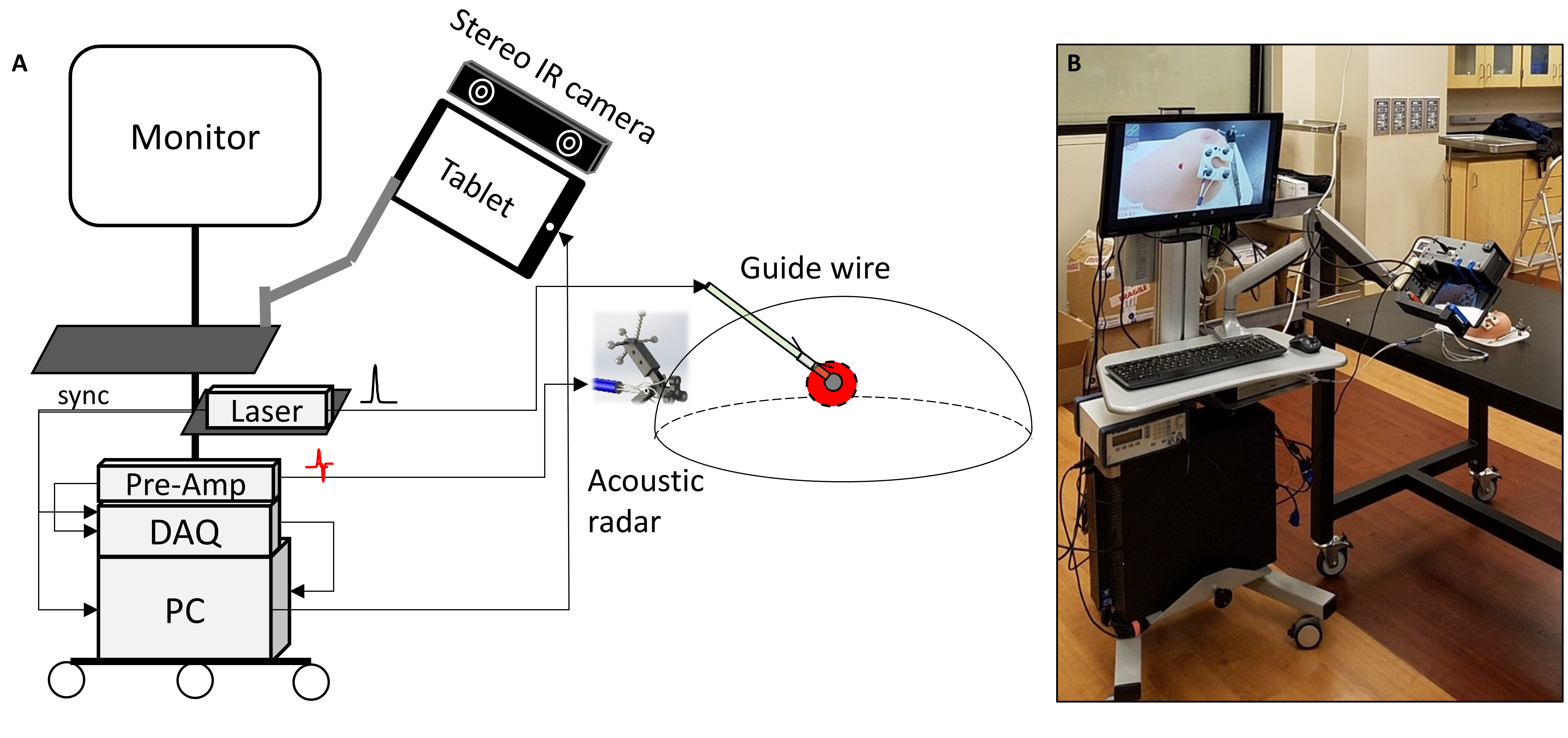

Supplement: Supplementary file 7 — Supplementary Figure s6(TIF 5038 kb) [file 41377_2018_6_MOESM7_ESM.tif]
