# Supplementary material for: A fiber optoacoustic guide with augmented reality for precision breast-conserving surgery
Source: Light Sci Appl. 2018 May 18;7:2. doi: 10.1038/s41377-018-0006-0 (PMC6107008; doi:10.1038/s41377-018-0006-0)
Supplement: Supplementary file 2 — Supplementary material(DOCX 3275 kb) [file 41377_2018_6_MOESM2_ESM.docx]

A Fiber Optoacoustic Guide with Augmented Reality for Precision Breast-Conserving Surgery

**Authors:** Lu Lan^1^, Yan Xia^2^, Rui Li^3^, Kaiming Liu^4^, Jieying Mai^3^, Jennifer Anne Medley^5^, Samilia Obeng-Gyasi^6^, Linda K. Han^7*^, Pu Wang^2*^, and Ji-Xin Cheng^1,8*^

**Affiliations:**

^1^Department of Biomedical Engineering, Boston University, 44 Cummington Mall, Boston, Massachusetts, 02215, USA

^2^Vibronix, Inc., 1281 Win Hentschel Blvd, West Lafayette, Indiana 47906, USA

^3^Department of Biomdeical Engineering, Purdue University, 206 S. Martin Jischke Dr., West Lafayette, Indiana 47907, USA

^4^Department of Precision Instrument, Tsinghua University, Beijing, 10084, China

^5^Department of Radiology and Imaging Sciences, Indiana University School of Medicine, 550 N. University Blvd, Indianapolis, Indiana 46202, USA

^6^Department of Surgery, Indiana University School of Medicine, 545 Barnhill Dr., Indianapolis, Indiana 46202, USA

^7^Parkview Cancer Institute, 11109 Parkview Plaza Dr., Fort Wayne, Indiana 46845, USA

^8^Department of Electrical & Computer Engineering, Boston University, 8 Saint Mary’s Street, Boston, Massachusetts, 02215, USA

^*^Corresponding author: Ji-Xin Cheng: jxcheng@bu.edu, Pu Wang: puwang@vibronixinc.com, Linda K. Han: linda.han@parkview.com

**One Sentence Summary:** A novel fiber optoacoustic guide with augmented reality to provide surgeons an intuitive visual guidance of the tumor location with sub-millimeter accuracy during the surgery, with which a board-certified breast cancer surgeon successfully demonstrated an excision experiment on a female human cadaver.

**Supplementary Material**

## Design of fiber optoacoustic guide.

In a real BCS operation, tumor excision is performed at an arbitrary position and angle with regard to the tumor site. Therefore, the optoacoustic signal generated by the fiber optoacoustic guide (FOG) should be detected across a wide angular range and over a large distance. The initial pressure generated by the pulsed laser excitation would be written as

|  | $P_{0}=\frac{\beta}{\kappa\rho C_{v}}\eta_{th}\mu_{a}F$ | (S1) |
| --- | --- | --- |

where $\beta$is the thermal expansion coefficient, $\kappa$ is the isothermal compressibility, $C_{v}$ is the specific heat capacity, $\eta_{th}$ is the heat conversion efficiency, $\mu_{a}$ is the optical absorption coefficient, and $F$ is the optical fluence [^1^](#_ENREF_1)^,^[^2^](#_ENREF_2) . To make the optoacoustic guide detectable in all directions and over a large distance range, we designed it from two main perspectives.

First, the distribution of light fluence, $F$, on the outer absorption layer needs to cover a wide angular range to enable a wide angular generation of optoacoustic signal. For an optical fiber, the angular range of light illumination is limited by its numerical aperture (NA). For example, the optical fiber with 0.22 NA can only spread light within an apex angle of 25.4$^{\circ}$ in air. We developed a diffuser sphere on the tip of fiber using composite of ZnO nanoparticles and epoxy to enable a wide angular light emission. The ZnO nanoparticles have a diameter of 100 nm, which is much smaller than the laser wavelength, thus enabling Raleigh scattering in all directions. Consequently, the incident light was scrambled in randomized directions, and a relatively uniform distributed angular light emission is produced at the tip of the FOG.

**Fig. S1 Measurement setup of angular light intensity distribution by diffuser sphere at the fiber tip and the results over different ZnO nano-particle concentrations**. **A** He-Ne laser light (632 nm) is coupled to optical fiber from the proximal end, and a photodiode (PD) is mounted on a motorized rotation stage (5 cm away from the rotation center) to measure the light intensity over different angles. Light intensity read-out is done by a digital oscilloscope. **B** Light intensity directivity map of the FOG guide with diffuser sphere composed of ZnO nano-particles with different concentrations (10%, 15% and 20%).

In general, the greater the number of scatters per unit volume inside the diffuser sphere, the more randomized and uniform angular distribution of light intensity would be obtained. On the other hand, scatters at high concentration would result in a decrease of light intensity for the conservation of total intensity input. To determine a good ZnO nanoparticle concentration in the light diffusion sphere, we measured the directivity of the light intensity by the diffusion spheres at 3 different ZnO concentrations (10%, 15% and 20% by weight) in experiments. **Fig. S1a** shows the experiment setup. A continuous 632 nm He-Ne Laser was applied as the light source, and the laser was coupled into the fiber through a fiber coupling lens (biconvex, f = 35 mm). A photodiode (DET 210, Thorlabs, Inc., NJ) was fixed at the motorized rotation stage (PRM1Z8, Thorlabs, Inc., NJ) at a distance of 5 cm to the rotation center, and then used to detect the light intensity at different angles. The step size of rotation angle was 10 degrees. A digital oscilloscope (DSO6014A, Agilent Technologies, CA) recorded the readout of the light intensity on the photodiode. Compared with the 10% and 20% concentration, the diffuser sphere with 15% ZnO nanoparticle concentration shows both a relatively high amplitude and uniform angular light distribution (**Fig. S1b**). Thus, ZnO nanoparticles of 15% concentration was chosen for later fiber optoacoustic guide fabrication.

**Fig. S2 Light absorption spectrum of the graphite and epoxy mixture solution**

Second, the intensity of initial optoacoustic signal needs to be strong enough to make the ultrasound signal detectable over a large distance. Therefore, materials with high optical absorption coefficient $\mu_{a}$, heat conversion efficiency$\eta_{th}$ and thermal expansion coefficient $\beta$ need to be applied as the outer absorption layer to transform light into large amplitude ultrasound. We chose a graphite and epoxy mixture to be the outer absorption layer on the diffuser sphere. Because: 1) graphite and epoxy are readily available, and 2) graphite has high optical absorption and heat transduction, and epoxy will then be driven to greatly expand to create acoustic wave, for graphite has 3 times higher thermal expansion coefficient (linear thermal coefficient: 45-65$\times{10}^{-3}$ m/K) than a typical metal (gold: 14$\times{10}^{-3}$ m/K). In experiments, the light absorption spectrum of the graphite (15% concentration by weight) and the epoxy mixture was measured, and nearly 100% light was absorbed over a broad wavelength range (**Fig. S2**), offering lots of freedom in choosing laser wavelength. Thus, absorption layer with over 15% concentration graphite composition could absorb all the light. Later, 30% concentration of graphite in the mixture was used in the experiment to increase the optoacoustic signal intensity.

In addition, the damage threshold of the FOG was tested by monitoring the generated optoacoustic signal with increasing energy input in experiments. The generated signal increased when the energy input increased to 1.3 mJ, and saturated till 2.75 mJ input was applied. The nano-composite sphere was observed to have damages when 2.75 mJ pulse energy was input (**Fig. S3**). Notably, here we developed the fiber optoacoustic guide with parameters and configurations above for a proof-of-concept omnidirectional optoacoustic emitter. Further improvements, such as better materials and design for the composite sphere [^3-5^](#_ENREF_3), can be applied to enhance its performance.

**Fig S3. Peak-to-peak amplitude of the generated optoacoustic (OA) signal with different laser pulse energy input.**

Lastly, we attached a short length stainless steel spring wire with heat shrink tube to grab it onto the FOG to prevent the migration of its tip in tissue. The two ends of the spring steel wire was slightly bent as hooks to grab the tissue. **Fig. S4** shows the process of how to release the FOG from an 18G introducer needle.

## **Fabrication of fiber optoacoustic guide**

**Fig S4. Design of hook sleeve on the fiber optoacoustic guide and the release process from an 18 gauge introducer needle.**

## The fabrication of fiber optoacoustic guide (FOG) was divided into two major steps. The first step was to form a light diffusion sphere on the fiber tip. The ZnO nanoparticles (~100 nm, Sigma-Aldrich, Inc., MO, USA) were mixed with epoxy at a concentration of 15% by weight in solution. One multimode optical fiber (200 µm core diameter, FT200EMT, Thorlabs, Inc., NJ, USA) with one of its tip polished was dipped about 100 µm below the surface of the mixture solution and then quickly pulled up. By sitting vertically at room temperature for 30 minutes, the solution on the tip cured and formed a light diffuser sphere. The diameter of the diffuser sphere was affected by the fiber diameter, the dipping depth and the submerging time of the fiber tip in the solution. The second step was to coat the diffuser with a graphite layer. Graphite powder (Dick Blick Holdings, Inc., IL, USA) was mixed with epoxy at a concentration of 30% by weight in solution. The fiber tip with the light diffuser sphere was then dipped in epoxy-graphite mixture solution with its entire diffuser sphere just submerged below the solution surface. Then, the fiber was quickly pulled up, and vertically placed to cure at room temperature for another 30 minutes.

## **Acoustic tracking using trilateration**

In geometry, trilateration is a process of determining absolute or relative locations by measurement of distances, using the geometry of circles, spheres or triangles. It has been widely used in practical applications in surveying and navigation, including global positioning system (GPS). In three-dimensional geometry, a target can be absolutely pinned down if its distances to four known detectors are obtained. If only distances to three detectors are known, we would solve two possible targets positioned symmetrically on two sides of the plane defined by the three detectors. Since the FOG tip is always inside breast tissue in our application, the FOG tip is going to be always on one particular side of the plane of the three transducers. So, the FOG tip can be located by just having its distances to three ultrasound transducers in our case. We mounted three identical ultrasound transducers on one 3D printed part. The three ultrasound transducers detect the time-of-flight acoustic signals, and send them to the host PC. Through detection of the delay of the acoustic signals to the excitation pulse, distances of the FOG tip to the three transducers are obtained. Using a customized trilateration software running on the host PC, the location of the FOG tip relative to the three transducers is solved.

## **Optical tracking of the acoustic radar using stereo vision**

Optical tracking of objects through stereo vision has been used in a broad spectrum of applications, such as robotic control, human motion capture, validation of computer vision and medical procedure planning. The process of determining a point in 3D space given its projections onto two, or more images is referred as triangulation in computer vision. Each point in an image corresponds to a line (projection line) in 3D space, i.e., all points on that line are projected to the same point on that image. If a point $x$ is captured by two cameras simultaneously, two projection lines can be plotted from the images on the two cameras and their intersecting point is the point$x$. Therefore, a target point in 3D space can be located with just a stereo camera. For a rigid object in 3D space, if over 4 points on it are located, its position and orientation in space can be solved. Using this method, we mount 5 infrared (IR) markers on the acoustic radar to track its position and orientation in the space with IR stereo camera.

The optical tracking of the acoustic radar is divided into two major steps. First, 5 infrared (IR) markers are mounted on the acoustic radar. To enable robust optical tracking of the acoustic radar, the markers mount to the acoustic radar is designed to be asymmetric, unique and with least self-obstruction in the camera view. Also, the marker mount is designed to be detachable to the acoustic radar for ease of use. The markers mount is later printed with high accuracy printer (Fortus 400mc 3D Systems, Stratasys Ltd., USA). A group of 5 IR reflective sphere markers (Precision Spheres with 3M 7610 Reflective Tape, NaturalPoint Inc., USA) are installed on the 3D printed mount in experiments. The IR markers, the mount and the acoustic radar all together assemble a rigid body which is clearly defined in the design file and remains unchanged in use.

Second, the rigid body formed by the IR markers and the acoustic radar is initialized to be trackable in the stereo vision system. A stereo vision system (OptiTrack V120 Duo, NaturalPoint, Inc., USA) with IR LED illumination and IR long pass filter on camera is used to initialize and track the marker group. Under IR illumination, the stereo vision system captures the IR reflective markers with dual cameras simultaneously and reconstructs their 3D positions. Based on the 3D positions of the markers, the stereo vision system creates a unique rigid body for the marker group on the acoustic radar and stores its profile. Such rigid body information is stored for real-time tracking later, and the initialization is finished. Then, the position and the orientation of the rigid body can be tracked in real-time (up to 120 frame/second) with 3D markers detection and a robust internal tracking algorithm by the stereo vision system. The tracking result of the acoustic radar is streamed to the host PC through local-area ethernet.

## **Transformation of the located tumor position into visual guidance on the AR display**

From the acoustic tracking above, the position of the FOG tip relative to the acoustic radar, $P_{RAD}$, is obtained. From the optical tracking method above, the position and orientation of the IR marker group mounted on the radar is obtained. Then combined with the known geometry of the marker-radar rigid body, we can transfer the FOG tip position from $P_{RAD}$ to the corresponding value, $P_{OPTI}$ in optical tracking coordinate. Next, we perform the camera calibration of the tablet-AR system to obtain the transformation matrix between the optical tracking coordinate and the AR rendering coordinate, $T_{AR}$, and the projection matrix of the tablet camera, $M_{C}$ (see ***supplementary material***). By applying the transformation matrix $T_{AR}$ to $P_{OPTI}$, the 3D position of the FOG tip in AR rendering coordinate,$P_{AR}$, is obtained. Lastly, with the projection matrix $M_{C}$, such AR rendering position is projected as a vivid cue on the AR display to achieve a merged view of the FOG tip in the operating scene.

## Design of the acoustic radar.

To enable trilateration of the FOG tip through acoustic detection, we developed an acoustic radar with 3 transducers placed with equal distance separated on a 3D printed mount. The accuracy of the trilateration is dependent on the spacing of the transducers. For a given axial / lateral shift of 0.39 mm (half wavelength of acoustic wave at 2 MHz), we calculated the detectable distance difference between 2 transducers (**Fig. S5A-B**). As the spacing of two transducers increases, the detectable distance difference by 2 transducers increases when the target has a given lateral shift (solid red line in **Fig. S5A**); the detectable distance difference decreases when the given shift is axial (solid red line in **Fig. S5B**). Therefore, the axial resolution of trilateration sets the upper boundary for the transducer spacing, and the lateral resolution of trilateration sets the lower boundary for the transducer spacing. 50MS/s data acquisition card was employed in the system to detect the time-of-flight acoustic signal, which offered a time resolution of 20 ns and thus a minimal detectable distance difference of 31 $\mu m$ = 1540 $m/s$ ($V_{s}$, speed of sound) * 20 ns. For the axial detection, the detectable difference is always larger than 0.3 mm when spacing of the transducer is in the range of 5 - 60 mm. Therefore, the spacing of the two transducers has to be larger than 7.4 mm to achieve a resolution of 0.39 mm for trilateration in our design. In our acoustic radar used, we mounted three identical transducers with 13.49 mm spacing to each other on a 3D printed mount (**Fig. S5C**). We chose the miniaturized transducer (2 mm diameter, XMS-310-B, Olympus, MA) for two reasons: 1) its compact size and 2) large receiving angle for its small diameter.

**Fig S5. Design of the acoustic radar. A,B** Detectable distance different of two transducers with different spacing, given a lateral and axial shift of 0.39 mm, respectively. Dash line represents the minimal detectable distance difference of 31 $\mu m$ by the DAQ system used. **C** Solidwork model of the designed acoustic radar with 13.95 mm spacing between each transducer.

## Design of the tablet-AR system

To minimize obstruction to surgical operations, we designed and 3D printed a compact mount to assemble the stereo IR camera (OptiTrack V120 Duo, NaturalPoint, Inc., USA) and the tablet (Shield Tablet K1, Nividia, USA) together. The tablet was placed closely below the center of the stereo IR camera baseline to achieve a largest shared field of view (FOV) among the three cameras. To transfer the optical tracking result into AR projection on the tablet-AR display, we performed the following camera calibration to obtain: 1) the transformation matrix between the optical tracking coordinate and the AR rendering coordinate, and 2) projection matrix of a position in 3D place onto the 2D tablet display.

## Camera calibration of the tablet-AR system

The camera calibration of the tablet-AR system is divided into two major steps.

The first step is the estimation of the transformation between the coordinate axes of stereo vision and the coordinate axes of tablet-AR rendering. A checkerboard holder with 4 IR reflective markers was designed and fabricated to capture stereo vision and tablet camera view simultaneously. The 4 IR markers and the printed 7X5 checkerboard were fixed on a two-layer acrylic board to be co-centered and well aligned. 4 mount bases were cut to place the 4 IR markers exactly on the 4 outmost corners of the checkboard by a laser cutter (Pro 24x16+ Laser, Full Spectrum Laser, USA). The tablet-AR system was placed at ~45 cm and ~55 cm from the checkerboard. While the tablet-AR system was kept still at each distance, the checkerboard was mounted on an articulating arm and adjusted to multiple heights and orientations. After each adjustment, the 3D positions of 4 IR markers were captured by the stereo vision system and the image of the checkerboard was captured by the tablet camera at the same time. The spatial orientation of the checkerboard can be calculated from the marker positions. Combined with the marker-base configuration, the 3D positions of 4 outmost corners on the checkerboard and the physical square size can be obtained. Then the 3D positions of all 35 corners can be further calculated while the 2D corner points were calculated through a robust checkerboard pattern detection method on the images captured [^6^](#_ENREF_6). Since the tablet and stereo IR system were fixed as a rigid body, we were able to pile data from multiple captures into a large set of 3D-2D (stereo vision-camera view) point correspondences. Then we fed the dataset into a Perspective-n-Point solver to obtain an optimal estimation of the rotational and translational offset between stereo vision system and tablet camera [^7^](#_ENREF_7)^,^[^8^](#_ENREF_8). The transformation combined with the projection matrix of tablet camera obtained in the first step enables us to project any object tracked by the stereo vision system onto the tablet-AR rendering scene.

The second step is the estimation of the parameters of tablet camera. A 7 x 5 checkerboard with physical square size of 30 mm each was used as the calibration pattern. The checkerboard was mounted on an articulating arm and multiple images of the checkerboard at various positions and orientations were taken. We used a standardized single camera calibration method based on pinhole camera model and a robust tool to estimate the intrinsic matrix and distortion vector of the tablet camera [^9^](#_ENREF_9). Then the projection matrix of tablet camera can be solved.

**Calculation of distance from the scalpel tip to the FOG tip**

With the acoustic tracking and optical tracking methods above (see ***Materials and Methods***), we can obtain the position of FOG tip, $P_{FOG}$ in the optical tracking coordinate. Likewise, we can calculate the position of the scalpel tip, $P_{SCP}$ from optical tracking of IR makers on the scalpel and the CAD profile of scalpel-marker rigid body. Then the distance between FOG tip and scalpel tip, $d_{FS}$ can be easily determined as $\left| P_{FOG}-P_{SCP} \right|$ in real-time.

## **Track the acoustic radar through an image target by head-mounted display (HMD) –AR system (Hololens)**

A 50 mm square image target is mounted on the acoustic radar for the tracking by our HMD-AR system (HoloLens). An image target mount with a 50 mm square frame was 3D printed and the image target printout with rich natural features is attached within the frame ^10^. Then the detachable image mount was assembled with the same acoustic radar used in tablet-AR system and they together form a rigid body, which is fully defined in the computer-aided design (CAD) file. The image target was captured by the RGB camera on the HMD, and its position and orientation in the HMD coordinate were calculated through a robust image tracking framework^11^. With the image target tracking result, we can transfer the acoustic tracking result into the HMD coordinate through the known geometry of image-radar rigid body. Then the tumor position was obtained and a sphere cue was rendered accordingly in the HMD.

## Design of compact transportable cart system for clinical translation

We integrated the compact laser, the DAQ system and the control unit into a compact transportable cart system for clinical translation (**Fig. S6**). The compact laser with its driver was placed on a side tray on the cart, which was connected to the FOG through a fiber connection to generate acoustic signals in tissue. The acoustic signals were detected by the acoustic radar and amplified by 3 identical pre-amplifiers (40 dB gain, 0.5 – 20 MHz, Model 5678, Olympus, USA), and then digitized by a digital oscilloscope (50MS/s, Oscar 14, DynamicSignals, USA). The active monitoring signal by the photodiode inside the laser was used to synchronize the entire system. A host PC processed the time-of-flight acoustic signals to triangulate the position of the FOG tip to the acoustic radar, and also received the position of the acoustic radar relative to the tablet through the stereo camera tracking. After coordinate transfer, the FOG tips’ location relative to the tablet view was calculated, and then sent to the table via wireless connection and project the position of the FOG tip on the tablet display. Also, the real-time tablet display was streamed to a monitor on the cart for easy access to the operator. The host PC and the DAQ system were all stacked at the bottom of the cart and the laser. The tablet with the stereo camera was held by an articulating arm mounted on the main tray of the mobile cart, and it could be moved around to view the operating scene at the operator’s preference.

**Fig S6. Compact integrated system on a mobile cart. A** Schematics of the modules of the integrated system on cart. Pre-amp: preamplifier for the ultrasonic signals. DAQ: data acquisition module, PC: host computer, sync: synchronization signal for all system modules from the active monitoring diode of the laser. **B** Photo of the cart system.

**Safety of the fiber optoacoustic guide wire**

As an active acoustic implant in the tissue, the acoustic wave generated at the FOG tip should not induce mechanical and thermal damage to the surrounding tissue. Mechanical index (MI) and thermal index (TI) is the metric of ultrasound beam’s ability to cause cavitation-related and thermal biological effects, respectively. The MI and TI of the FOG is estimated to be < 0.03 and < 0.28 (see session below), respectively, which are both over 20 times lower than the safety standard by the FDA [^10^](#_ENREF_10). Therefore, the risk of causing mechanical and thermal damage to tissue is quite low by our FOG.

## **Estimation of the mechanical index and thermal index of the acoustic wave by the fiber optoacoustic guide**

The noise equivalent pressure (NEP) for a piezoelectric transducer of 50 MHz bandwidth with a detector area of 30 ${mm}^{2}$ is ~ 77 Pa at room temperature [^11^](#_ENREF_11). The NEP is described as:

|  | $NEP\left( f \right)= \sqrt{F_{n}k_{B}TZ_{a}/[A\eta(f)]}$ | (S2) |
| --- | --- | --- |

Where $\eta(f)$ is the detector efficiency at frequency$f$, $k_{B}$ is the Boltzmann constant, $T$ is the absolute temperature of the medium, $Z_{a}$ is the characteristic acoustic impedance of the medium, $A$ is the detector area, and low-noise amplifier typically exhibit $F_{n}\approx2$ [^12^](#_ENREF_12). Given the 10 MHz transducer used has 2 mm diameter and $\eta\left( f \right)$ of about 0.05, the NEP in our experiment is ~ 100 Pa. With a maximum SNR of 47 dB measured in experiments, the pressure of the acoustic wave generated by our FOG is estimated to be ~ 22 kPa, which is less than 50 kPa. Therefore, the mechanical index (MI) is estimated to be less than 0.025, through following equation:

|  | $MI=p_{r,3}(z_{sp})/(f^{1/2})$ | (S3) |
| --- | --- | --- |

Where $p_{r,3}(z_{sp})$ is the peak rarefractional pressure (in MPa) derated by 0.3 $\mathrm{dB}\cdot{cm}^{-1}\cdot{MHz}^{-1}$, $f$ is the center frequency in (MHz). So, our FOG has a MI of < 0.03, which is over 20 times lower than the predicted value of 0.83 when cavitation theoretically occurs.

As for thermal index, it is associated with the acoustic power $W_{\alpha}$and the acoustic power $W_{deg}$ required to raise the tissue temperature by 1 $℃$ , as shown below .

|  | $TI=\frac{W_{\alpha}}{W_{deg}}$ | (S4) |
| --- | --- | --- |

For an acoustic wave of 2.25MHz frequency and 2.5MPa peak negative pressure operating at 500 Hz repetition rate, its mechanic index (MI) is about 1.6 and thermal index (TI) is about 0.28 [^13^](#_ENREF_13) . Our FOG generates acoustic wave of comparable frequency and repetition rate, but with lower pressure, i.e. lower acoustic power. Thus, the acoustic wave emitted by our FOG is less than 0.28.

**Supplementary Videos**

**Movie S1.** Visualization of fiber optoacoustic guide tip in the breast on female cadaver with the tablet-AR system

**Movie S2.** Visualization of fiber optoacoustic guide tip in the breast on female cadaver with the Hololens-AR system

**Movie S3.** Implantation and confirmation of the biopsy clip and the fiber optoacoustic guide under ultrasound imaging guidance

**Movie S4.** Excision experiment on the female cadaver

**References:**

1 Wang, L. V. Tutorial on photoacoustic microscopy and computed tomography. *IEEE Journal of Selected Topics in Quantum Electronics* **14**, 171-179 (2008).

2 Wang, L. V. *Photoacoustic imaging and spectroscopy*. (CRC press, 2009).

3 Baac, H. W., Ok, J. G., Lee, T. & Guo, L. J. Nano-structural characteristics of carbon nanotube–polymer composite films for high-amplitude optoacoustic generation. *Nanoscale* **7**, 14460-14468 (2015).

4 Baac, H. W. *et al.* Carbon-nanotube optoacoustic lens for focused ultrasound generation and high-precision targeted therapy. *Scientific reports* **2**, 989 (2012).

5 Lee, T., Li, Q. & Guo, L. J. Out-coupling of longitudinal photoacoustic pulses by mitigating the phase cancellation. *Scientific reports* **6**, 21511 (2016).

6 Gao, Xiao-Shan, Xiao-Rong Hou, Jianliang Tang, and Hang-Fei Cheng. "Complete solution classification for the perspective-three-point problem." IEEE transactions on pattern analysis and machine intelligence 25, 930-943 (2003).

7 Gao, X.-S., Hou, X.-R., Tang, J. & Cheng, H.-F. Complete solution classification for the perspective-three-point problem. *IEEE transactions on pattern analysis and machine intelligence* **25**, 930-943 (2003).

8 Fischler, M. A. & Bolles, R. C. Random sample consensus: a paradigm for model fitting with applications to image analysis and automated cartography. *Communications of the ACM* **24**, 381-395 (1981).

9 Geiger, A., Moosmann, F., Car, Ö. & Schuster, B. in *Robotics and Automation (ICRA), 2012 IEEE International Conference on.* 3936-3943 (IEEE).

10 Vuforia library, Optimizing Target Detection and Tracking Stability, <https://library.vuforia.com/articles/Solution/Optimizing-Target-Detection-and-Tracking-Stability>

11 Vuforia library, How To Use Object Recognition in Unity, <https://library.vuforia.com/articles/Solution/How-To-Use-Object-Recognition-in-Unity>

12 Zhang, Z. A flexible new technique for camera calibration. *IEEE Transactions on pattern analysis and machine intelligence* **22**, 1330-1334 (2000).

13 Food & Administration, D. Guidance for Industry and FDA Staff Information for Manufacturers Seeking Marketing Clearance of Diagnostic Ultrasound Systems and Transducers. *Silver Spring: US FDA* (2008).

14 Yao, J. & Wang, L. V. Sensitivity of photoacoustic microscopy. *Photoacoustics* **2**, 87-101 (2014).

15 Winkler, A. M., Maslov, K. & Wang, L. V. Noise-equivalent sensitivity of photoacoustics. *Journal of biomedical optics* **18**, 097003-097003 (2013).
